# Tris(ethyl­enediamine)zinc(II) hexa­fluorido­silicate

**DOI:** 10.1107/S1600536809045693

**Published:** 2009-11-07

**Authors:** Yang Li, Qi Shi, Alexandra M. Z. Slawin, J. Derek Woollins, Jinxiang Dong

**Affiliations:** a Department of Chemistry, University of St Andrews, St Andrews KY16 9ST, Scotland; b Research Institute of Special Chemicals, Taiyuan University of Technology, Taiyuan 030024, ShanXi, People’s Republic of China

## Abstract

The title compound, [Zn(C_2_H_8_N_2_)_3_](SiF_6_), was synthesized ionothermally using choline chloride–imidazolidone as solvent and template provider. In the crystal structure, the anions and cations are located on special positions of site symmetry 3.2 and show a typical octa­hedral geometry. The Zn^II^ ion is coordinated by six N atoms from three ethyl­enediamine mol­ecules. The crystal structure displays weak hydrogen bonding between [SiF_6_]^2−^ anions and the ethyl­enediamine NH hydrogen atoms.

## Related literature

For related structures, see: Ray *et al.* (1973 ▶); Bernhardt & Riley (2003 ▶); Cernak *et al.* (1984 ▶); Emsley *et al.* (1989 ▶); Cheng *et al.* (2008 ▶).
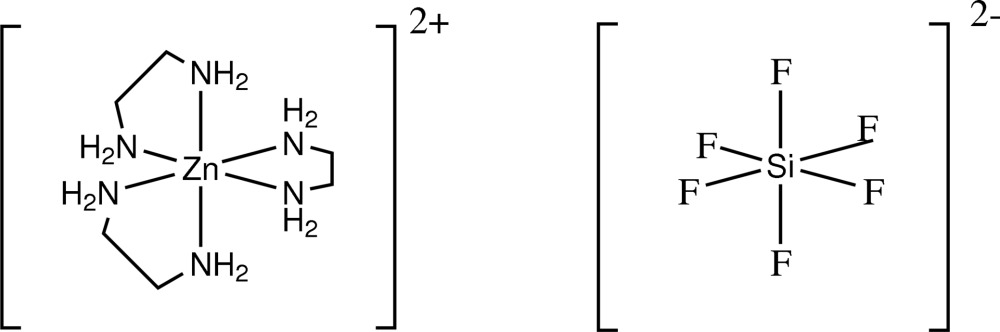


## Experimental

### 

#### Crystal data

[Zn(C_2_H_8_N_2_)_3_](SiF_6_)
*M* *_r_* = 387.77
Hexagonal, 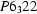

*a* = 9.192 (2) Å
*c* = 9.755 (3) Å
*V* = 713.8 (3) Å^3^
*Z* = 2
Mo *K*α radiation
μ = 1.87 mm^−1^
*T* = 93 K
0.10 × 0.10 × 0.10 mm

#### Data collection

Rigaku Mercury CCD diffractometer
Absorption correction: multi-scan (*CrystalClear*; Rigaku, 2004 ▶) *T*_min_ = 0.835, *T*_max_ = 0.835
4809 measured reflections
534 independent reflections
499 reflections with *I* > 2σ(*I*)
*R*_int_ = 0.045

#### Refinement

*R*[*F* ^2^ > 2σ(*F* ^2^)] = 0.027
*wR*(*F* ^2^) = 0.059
*S* = 1.11
534 reflections
32 parameters
H-atom parameters constrained
Δρ_max_ = 0.51 e Å^−3^
Δρ_min_ = −0.38 e Å^−3^
Absolute structure: Flack (1983 ▶), 177 Friedel pairs
Flack parameter: 0.01 (3)

### 

Data collection: *CrystalClear* (Rigaku, 2004 ▶); cell refinement: *CrystalClear*; data reduction: *CrystalClear*; program(s) used to solve structure: *SHELXS97* (Sheldrick, 2008 ▶); program(s) used to refine structure: *SHELXL97* (Sheldrick, 2008 ▶); molecular graphics: *PLATON* (Spek, 2009 ▶); software used to prepare material for publication: *SHELXTL* (Sheldrick, 2008 ▶).

## Supplementary Material

Crystal structure: contains datablocks I, global. DOI: 10.1107/S1600536809045693/bt5123sup1.cif

Structure factors: contains datablocks I. DOI: 10.1107/S1600536809045693/bt5123Isup2.hkl

Additional supplementary materials:  crystallographic information; 3D view; checkCIF report

## Figures and Tables

**Table 1:** Hydrogen-bond geometry (Å, °)

*D*—H⋯*A*	*D*—H	H⋯*A*	*D*⋯*A*	*D*—H⋯*A*
N1—H1*A*⋯F1^i^	0.92	2.26	3.113 (3)	155
N1—H1*A*⋯F1^ii^	0.92	2.49	3.239 (3)	139
N1—H1*B*⋯F1^iii^	0.92	2.25	3.153 (3)	166

Symmetry codes: (i) 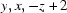; (ii) 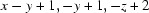; (iii) 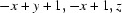.

**Fig. 1.**
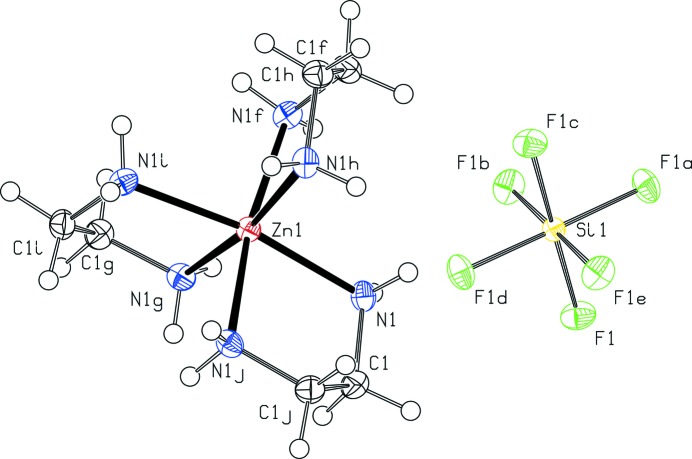
The molecular structure of the title compound with displacement ellipsoids drawn at the 50% probability level.

## supplementary crystallographic information

### Comment

A large number of salts with the general formula MG_6_L*R*_6_, where *M* is a bivalent metal, G may be water or ammonia, *L* is a quadrivalent element like Si, Sn, Ti or Zr, and *R* may be Cl, F or CN, (Ray *et al.*, 1973), were studied. We report a similar type of the title salt containing organic molecules. The molecule of the title salt, shown in Fig. 1, consists of one Zn(C_2_N_2_H_8_)_3_ cation and one SiF_6_ anion. The coordination of ZnII centers through bridging-bidentate ethylenediamine groups forms a wind-stick-like cluster. The Zn(C_2_N_2_H_8_)_3_ cluster and SiF_6_ octahedra are stacked alternately along the threefold axis in approximately CsCl-type packing.

### Experimental

A typical synthetic procedure for Zn(C_2_N_2_H_8_)_3_.SiF_6_ was as follows: a Teflon-lined autoclave (volume 15 ml) was charged with the ionic liquid [composed of choline chloride (1630 mg, 11.4 mmol) and imidazolidone (2.045 g, 22.8 mmol)], zinc acetate (168 mg, 0.74 mmol), NH_4_F (71 mg, 1.85 mmol), and silica (49 mg, 0.74 mmol) and heated in an oven at 180 °C for 3 days. Ethylenediamine(C_2_N_2_H_8_), derived from decomposition of the imidazolidone component of the deep-eutectic solvent (DES) itself, is delivered to the synthesis. The synthesized samples were washed with distilled water in an ultrasonic bath, then washed with acetone, and dried at room temperature in air. The colorless crystals of the title salt were abtained with suitable size for single-crystal X-ray analysis.

### Refinement

All H atoms were fixed geometrically (C—H = 0.99 Å, N—H = 0.92 Å) and treated as riding with *U*_iso_(H) = 1.2Ueq of the parent atom.

### Crystal data

**Table:**

[Zn(C_2_H_8_N_2_)_3_](SiF_6_)	*D*_x_ = 1.804 Mg m^−^^3^
*M**_r_* = 387.77	Mo *K*α radiation, λ = 0.71073 Å
Trigonal, *P*6_3_22	Cell parameters from 1402 reflections
Hall symbol: P 6c 2c	θ = 6.6–54.6°
*a* = 9.192 (2) Å	µ = 1.87 mm^−^^1^
*c* = 9.755 (3) Å	*T* = 93 K
*V* = 713.8 (3) Å^3^	Prism, colorless
*Z* = 2	0.10 × 0.10 × 0.10 mm
*F*(000) = 400	

### Data collection

**Table:**

Rigaku Mercury CCD diffractometer	534 independent reflections
Radiation source: rotating anode	499 reflections with *I* > 2σ(*I*)
confocal	*R*_int_ = 0.045
Detector resolution: 0.83 pixels mm^-1^	θ_max_ = 27.4°, θ_min_ = 3.3°
ω scans	*h* = −10→11
Absorption correction: multi-scan (*CrystalClear*; Rigaku, 2004)	*k* = −10→11
*T*_min_ = 0.835, *T*_max_ = 0.835	*l* = −11→9
4809 measured reflections	

### Refinement

**Table:**

Refinement on *F*^2^	Secondary atom site location: difference Fourier map
Least-squares matrix: full	Hydrogen site location: inferred from neighbouring sites
*R*[*F*^2^ > 2σ(*F*^2^)] = 0.027	H-atom parameters constrained
*wR*(*F*^2^) = 0.059	*w* = 1/[σ^2^(*F*_o_^2^) + (0.0158*P*)^2^ + 0.5912*P*] where *P* = (*F*_o_^2^ + 2*F*_c_^2^)/3
*S* = 1.11	(Δ/σ)_max_ < 0.001
534 reflections	Δρ_max_ = 0.51 e Å^−^^3^
32 parameters	Δρ_min_ = −0.38 e Å^−^^3^
0 restraints	Absolute structure: Flack (1983), 177 Friedel pairs
Primary atom site location: structure-invariant direct methods	Flack parameter: 0.01 (3)

### Special details

**Table:**

Geometry. All e.s.d.'s (except the e.s.d. in the dihedral angle between two l.s. planes) are estimated using the full covariance matrix. The cell e.s.d.'s are taken into account individually in the estimation of e.s.d.'s in distances, angles and torsion angles; correlations between e.s.d.'s in cell parameters are only used when they are defined by crystal symmetry. An approximate (isotropic) treatment of cell e.s.d.'s is used for estimating e.s.d.'s involving l.s. planes.
Refinement. Refinement of *F*^2^ against ALL reflections. The weighted *R*-factor *wR* and goodness of fit *S* are based on *F*^2^, conventional *R*-factors *R* are based on *F*, with *F* set to zero for negative *F*^2^. The threshold expression of *F*^2^ > σ(*F*^2^) is used only for calculating *R*-factors(gt) *etc*. and is not relevant to the choice of reflections for refinement. *R*-factors based on *F*^2^ are statistically about twice as large as those based on *F*, and *R*- factors based on ALL data will be even larger.

### Fractional atomic coordinates and isotropic or equivalent isotropic displacement parameters (Å^2^)

**Table:**

	*x*	*y*	*z*	*U*_iso_*/*U*_eq_	
Si1	0.3333	0.6667	0.7500	0.0135 (3)	
F1	0.48288 (18)	0.6657 (2)	0.85081 (14)	0.0250 (3)	
Zn1	0.6667	0.3333	0.7500	0.01508 (18)	
N1	0.8544 (2)	0.5434 (2)	0.87149 (19)	0.0191 (4)	
H1A	0.8277	0.5243	0.9631	0.023*	
H1B	0.9584	0.5539	0.8589	0.023*	
C1	0.8584 (4)	0.6997 (3)	0.8277 (2)	0.0222 (5)	
H2A	0.9655	0.7986	0.8566	0.027*	
H2B	0.7651	0.7070	0.8716	0.027*	

### Atomic displacement parameters (Å^2^)

**Table:**

	*U*^11^	*U*^22^	*U*^33^	*U*^12^	*U*^13^	*U*^23^
Si1	0.0151 (4)	0.0151 (4)	0.0102 (7)	0.0076 (2)	0.000	0.000
F1	0.0260 (7)	0.0352 (8)	0.0183 (7)	0.0186 (7)	−0.0057 (6)	−0.0011 (7)
Zn1	0.0169 (2)	0.0169 (2)	0.0115 (3)	0.00843 (11)	0.000	0.000
N1	0.0185 (10)	0.0240 (11)	0.0137 (11)	0.0097 (9)	−0.0018 (8)	0.0003 (8)
C1	0.0234 (13)	0.0195 (11)	0.0225 (12)	0.0099 (13)	−0.0031 (12)	−0.0037 (8)

### Geometric parameters (Å, °)

**Table:**

Si1—F1^i^	1.6938 (13)	Zn1—N1^v^	2.186 (2)
Si1—F1^ii^	1.6938 (13)	Zn1—N1^viii^	2.1863 (19)
Si1—F1^iii^	1.6938 (14)	Zn1—N1^ix^	2.1863 (19)
Si1—F1^iv^	1.6938 (14)	N1—C1	1.482 (3)
Si1—F1	1.6938 (13)	N1—H1A	0.9200
Si1—F1^v^	1.6938 (13)	N1—H1B	0.9200
Zn1—N1^vi^	2.1863 (19)	C1—C1^viii^	1.523 (4)
Zn1—N1^vii^	2.1863 (19)	C1—H2A	0.9900
Zn1—N1	2.186 (2)	C1—H2B	0.9900
			
F1^i^—Si1—F1^ii^	90.69 (10)	N1^vi^—Zn1—N1^viii^	93.40 (7)
F1^i^—Si1—F1^iii^	89.68 (7)	N1^vii^—Zn1—N1^viii^	170.67 (11)
F1^ii^—Si1—F1^iii^	89.95 (10)	N1—Zn1—N1^viii^	80.19 (10)
F1^i^—Si1—F1^iv^	89.95 (10)	N1^v^—Zn1—N1^viii^	93.40 (7)
F1^ii^—Si1—F1^iv^	89.68 (7)	N1^vi^—Zn1—N1^ix^	170.67 (11)
F1^iii^—Si1—F1^iv^	179.47 (11)	N1^vii^—Zn1—N1^ix^	93.40 (7)
F1^i^—Si1—F1	179.47 (11)	N1—Zn1—N1^ix^	93.40 (7)
F1^ii^—Si1—F1	89.68 (7)	N1^v^—Zn1—N1^ix^	80.19 (10)
F1^iii^—Si1—F1	90.69 (10)	N1^viii^—Zn1—N1^ix^	93.74 (11)
F1^iv^—Si1—F1	89.68 (7)	C1—N1—Zn1	109.01 (14)
F1^i^—Si1—F1^v^	89.68 (7)	C1—N1—H1A	109.9
F1^ii^—Si1—F1^v^	179.47 (11)	Zn1—N1—H1A	109.9
F1^iii^—Si1—F1^v^	89.68 (7)	C1—N1—H1B	109.9
F1^iv^—Si1—F1^v^	90.69 (10)	Zn1—N1—H1B	109.9
F1—Si1—F1^v^	89.95 (10)	H1A—N1—H1B	108.3
N1^vi^—Zn1—N1^vii^	80.19 (10)	N1—C1—C1^viii^	109.52 (17)
N1^vi^—Zn1—N1	93.74 (11)	N1—C1—H2A	109.8
N1^vii^—Zn1—N1	93.40 (7)	C1^viii^—C1—H2A	109.8
N1^vi^—Zn1—N1^v^	93.40 (7)	N1—C1—H2B	109.8
N1^vii^—Zn1—N1^v^	93.74 (11)	C1^viii^—C1—H2B	109.8
N1—Zn1—N1^v^	170.67 (11)	H2A—C1—H2B	108.2
			
N1^vi^—Zn1—N1—C1	106.99 (17)	N1^ix^—Zn1—N1—C1	−79.03 (19)
N1^vii^—Zn1—N1—C1	−172.64 (16)	Zn1—N1—C1—C1^viii^	−39.9 (3)
N1^viii^—Zn1—N1—C1	14.19 (13)		

Symmetry codes: (i) −*x*+*y*, *y*, −*z*+3/2; (ii) −*y*+1, *x*−*y*+1, *z*; (iii) *x*, *x*−*y*+1, −*z*+3/2; (iv) −*x*+*y*, −*x*+1, *z*; (v) −*y*+1, −*x*+1, −*z*+3/2; (vi) *x*, *x*−*y*, −*z*+3/2; (vii) −*x*+*y*+1, −*x*+1, *z*; (viii) −*x*+*y*+1, *y*, −*z*+3/2; (ix) −*y*+1, *x*−*y*, *z*.

### Hydrogen-bond geometry (Å, °)

**Table:**

*D*—H···*A*	*D*—H	H···*A*	*D*···*A*	*D*—H···*A*
N1—H1A···F1^x^	0.92	2.26	3.113 (3)	155
N1—H1A···F1^xi^	0.92	2.49	3.239 (3)	139
N1—H1B···F1^vii^	0.92	2.25	3.153 (3)	166

Symmetry codes: (x) *y*, *x*, −*z*+2; (xi) *x*−*y*+1, −*y*+1, −*z*+2; (vii) −*x*+*y*+1, −*x*+1, *z*.
